# Does refining an intervention based on participant feedback increase acceptability? An experimental approach

**DOI:** 10.1186/s12889-023-16344-w

**Published:** 2023-08-22

**Authors:** Chris Keyworth, Leah Quinlivan, Jessica Z. Leather, Rory C. O’Connor, Christopher J. Armitage

**Affiliations:** 1 School of Psychology, University of Leeds, Leeds, LS2 9JT UK; 2 NIHR Greater Manchester Patient Safety Translational Research Centre, Manchester Academic Health Science Centre, The University of Manchester, Manchester, M13 9PL UK; 3 Manchester Centre for Health Psychology, School of Health Sciences, The University of Manchester, Manchester, M13 9PL UK; 4 Suicidal Behaviour Research Laboratory, Institute of Health and Wellbeing, University of Glasgow, Glasgow, UK; 5 Manchester University NHS Foundation Trust, Manchester Academic Health Science Centre, Manchester, M13 9PL UK

**Keywords:** Acceptability, Experimental, Intervention design, Mixed methods

## Abstract

**Background:**

Participant feedback is an important consideration for increasing intervention acceptability, yet whether incorporating such feedback actually improves acceptability is rarely tested.

**Purpose:**

The present study describes a theory-based approach to assessing whether refining an intervention based on participant feedback increases acceptability.

**Methods:**

Three hundred and ninety-three UK adults who had previously self-harmed were exposed to the same intervention at baseline and, six months later, were randomly allocated to receive either: (a) the same version of the intervention (control group), or (b) a version of the intervention that had been refined following participant feedback (experimental group). The main outcome measure was acceptability ratings for each of the seven domains specified in the Theoretical Framework of Acceptability (TFA).

**Results:**

Mixed ANOVAs, with control versus experimental group as the between-participants factor and time (baseline versus follow-up) as the within participants factor showed no significant changes in acceptability.

**Conclusions:**

The null effects reported here imply that participants found both the original and modified versions of the intervention equally acceptable, and that our process of refining an intervention based on participant feedback did not impact on acceptability. Nevertheless, we have operationalised a robust approach for examining whether participant feedback impacts on the acceptability of an intervention. Further research is required to understand better how participant feedback should be incorporated into the development of healthcare interventions.

**Supplementary Information:**

The online version contains supplementary material available at 10.1186/s12889-023-16344-w.

## Background

Various factors affect the successful implementation of an intervention, such as its feasibility, desirability, and perceived appropriateness [1, 2]. Intervention acceptability in particular is an important consideration in the design, implementation, and evaluation of healthcare interventions [3, 4]. The UK Medical Research Council (MRC) guidelines for developing and evaluating complex interventions recommend assessing intervention acceptability (a key consideration for intervention design and refinement) by engaging potential intervention users to inform the refinements to interventions [2]. The likelihood of successful implementation and subsequent effectiveness is dependent upon perceptions of acceptability [4, 5]. For example, interventions perceived as acceptable by those delivering and/or receiving them are more likely to result in favourable outcomes including adherence to treatment programmes [6], or support for public health policy [5].

Involving people in the design and modification of interventions is recognised as an important stage in ensuring acceptability [1]. Key principles include encouraging researchers to view intervention development as iterative cycles of development to refine an intervention using feedback from people outside the research team throughout the process. Coproduction can involve researchers working together with the public or those with lived experience from the start of a research programme, sharing power and responsibility, and generating knowledge [7]. There is evidence across a broad range of healthcare settings that coproducing interventions with key stakeholders may increase perceptions of intervention acceptability [8, 9]. Additionally, coproduction, for example, may involve people with lived experience providing feedback on an existing intervention with the overarching aim of making the intervention more acceptable and feasible [1]. This could involve stakeholders and intervention developers generating ideas about content, format, style and delivery of interventions [7]. However, there are two potential limitations of this approach.

The first limitation concerns the lack of study methods explicitly testing the acceptability of a modified intervention following participant feedback. This is important because, to our knowledge, no experimental studies have been conducted that evaluate whether changes made to an intervention in response to participant feedback elicited improvements in acceptability.

The second limitation concerns the lack of use of theoretical frameworks to guide the investigation of acceptability. The Theoretical Framework of Acceptability (TFA) [4, 10] is an established guide to assess the acceptability of interventions. It defines acceptability as “a multi-faceted construct that reflects the extent to which people delivering or receiving a healthcare intervention consider it to be appropriate, based on anticipated or experienced cognitive and emotional responses to the intervention” (p.4) [4]. The TFA comprises seven domains: (1) affective attitude (how individuals feel about taking part in an intervention), (2) burden (the amount of effort required to engage with an intervention), (3) perceived effectiveness (whether individuals perceive an intervention as likely to achieve its purpose), (4) ethicality (the extent to which an intervention fits with individuals’ personal values), (5) intervention coherence (whether individuals understand an intervention and how it works), (6) opportunity costs (what is given up, such as time, to take part in an intervention), and (7) self-efficacy (how confident individuals are doing the intervention). The advantage of using the TFA, as opposed to more general approaches to evaluating acceptability, is that the TFA allows a more systematic assessment of intervention acceptability that is comparable across interventions, and enables researchers to target specific TFA domains in future iterations of interventions (e.g. addressing perceived burden of interventions) [11].

## Aims

The aims of the present study were to operationalise an approach to intervention acceptability that: (a) ensures that a large representative sample of people with lived experience is involved in the process of refining an intervention to increase acceptability; and (b) adopts a theory-driven, experimental approach to evaluating whether refining an intervention based on participant feedback increases acceptability.

### Methods

#### Overview

Ethical approval was obtained from The University of Manchester Research Ethics Committee (ref: 2020-8446-15312). All methods were performed in accordance with the relevant guidelines and regulations (Declaration of Helsinki). The study was conducted in three phases as part of a larger six-month follow-up study examining the effectiveness of an intervention for reducing self-harm (ClinicalTrials.gov Identifier: NCT04420546). In phase 1, we focused on intervention development, and phase 2 focused on developing and testing an experimental approach to evaluating whether refining an intervention based on participant feedback increases acceptability. Phase 3 is currently ongoing, and is focused on evaluating the effectiveness of the intervention.

### Phase 1: intervention development

#### Development of the volitional help sheet for self-harm

The intervention is based on the concept of implementation intentions [12], which are “if-then” plans that help people to link a critical situation (i.e. “if”) with an appropriate response (i.e. “then”). If-then plans work by making automatic links [13] in memory between a critical situation (“If I am tempted to self-harm when I want to get some attention…”) and an appropriate response (“…then I will do something else instead of self-harming”). The volitional help sheet (VHS) is a tool designed to assist with the formation of implementation intentions to reduce self-harm, and has previously been shown to be effective in reducing self-harm in people recently admitted to the hospital for self-harm [14].

Participant suggestions for refining the intervention were coded to the specific constructs of the TFA as part of our qualitative analyses conducted during our prior intervention development work on the original intervention [15, 16]. Briefly, a directed content analysis approach was used to identify and categorise instances of the TFA domains. To increase the trustworthiness of the data, two authors were involved in the coding process (CK and CJA), with any areas of contention discussed and agreed upon accordingly. A more detailed overview of the coding process is presented elsewhere [13]. Based on the feedback provided by three-hundred and forty participants, three specific amendments were made based on open-ended comments. Firstly, participants described how it would be helpful to emphasise to participants before completing the volitional help sheet that not all situations and solutions were relevant to everyone. Therefore, changes were made to the instructions of the volitional help sheet to further increase clarity and understanding (in line with the *intervention coherence* domain of the TFA). Secondly, participants suggested that the VHS would be improved if they could enter their own situations and solutions if the statements were not applicable to them. Therefore, open-text fields were added to the volitional help sheet (in line with the *ethicality* domain of the TFA). Thirdly, participants made useful suggestions about improving the formatting and layout of the intervention webpage. Therefore, prior to distribution, the research team ensured the intervention could be viewed effectively on both a desktop computer and a mobile device (in line with *burden* domain of the TFA).

**Fig. 1:**
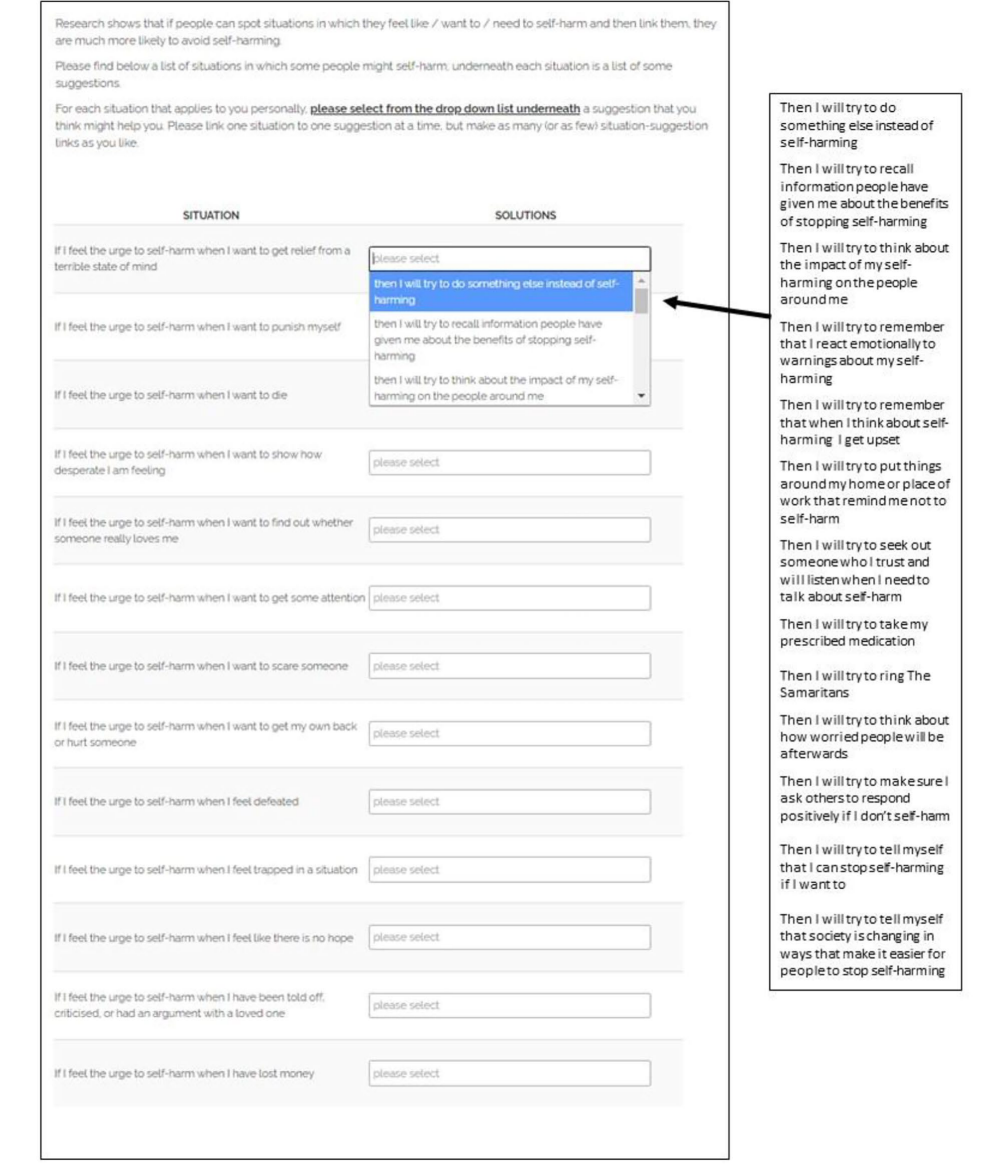
Volitional help sheet for self-harm (control condition)

**Fig. 2:**
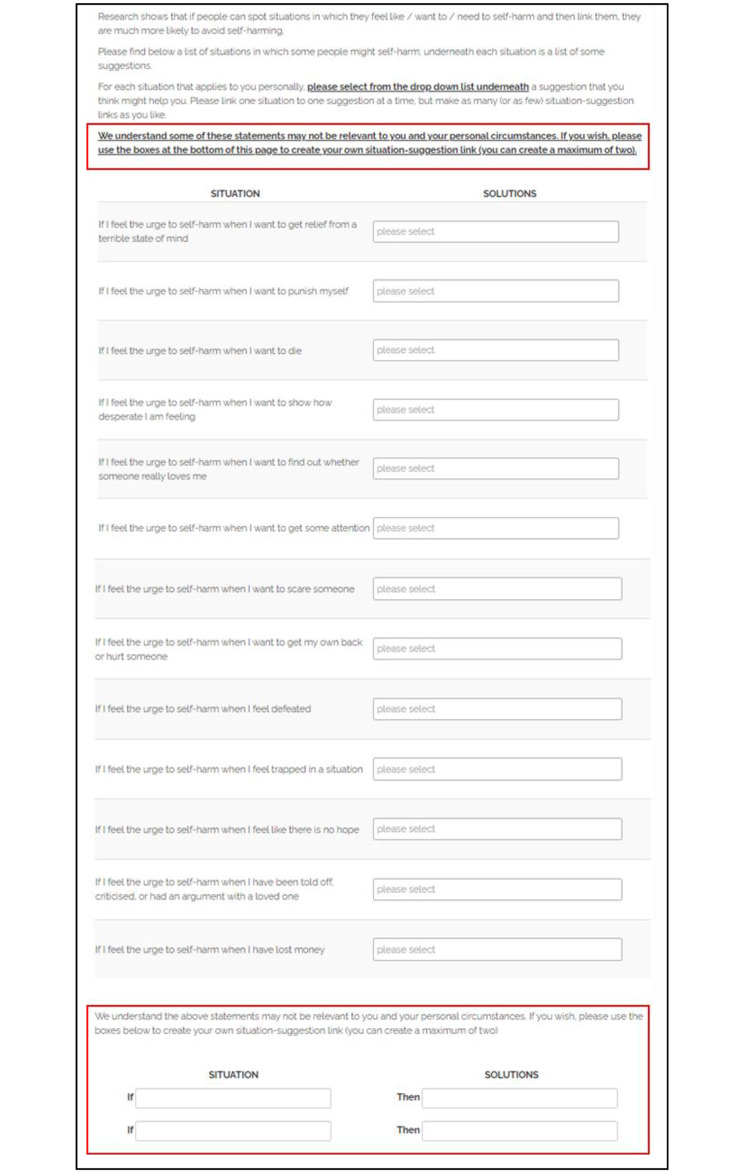
Volitional help sheet for self-harm (experimental condition)

The original and modified versions are presented in Figs. 1 and 2 respectively, with modifications highlighted in red (see Fig. 2). The intervention was presented on a single webpage, with a list of situations alongside which participants could choose an appropriate response from a drop-down menu for each critical situation. Specific modifications were made based on the feedback provided by participants at baseline, via open ended comments in line with the seven TFA domains [15].

### Phase 2: main study

#### Participants

A national community sample of people in the UK who had previously self-harmed were recruited via a survey panel company (YouGov), as part of a larger study (ClinicalTrials.gov Identifier: NCT04420546). Participants were incentivised in line with YouGov’s points system (respondents accumulate points for taking part in surveys, which can then be exchanged for cash or entry into a prize draw). A screening question was asked to ensure that the final sample contained people with a prior history of self-harm: “Have you ever intentionally hurt yourself/self-harmed?” Response options were “yes, I have,” “no, I have not,” or “prefer not to say.” The final sample was based on respondents answering, “yes, I have.”

### Design

A mixed-measures design was employed with one between-participants factor (*condition*: modified intervention versus original intervention) and one within-participants factor (*time*: pre-randomisation versus six-month follow-up). The primary outcome measure was acceptability, divided into the seven TFA domains: *Affective Attitude*, *Burden*, *Ethicality*, *Self-efficacy*, *Opportunity Costs*, *Intervention Coherence*, and *Perceived Effectiveness*.

#### Procedure

At baseline, after participants gave informed consent, all were presented with the original intervention aimed at reducing self-harm. Participants were then randomised via online survey software to receive, at six-month follow-up, either: (a) the same version of the intervention, or (b) a version of the intervention that had been changed following participant feedback. Whilst participants were not expected to use the intervention in the period between baseline and follow-up, the volitional help sheet is intended to provide people with a means of responding to critical situations (where the urge to self-harm may be heightened), with automatic coping plans that were formed during the baseline period.

### Measures

#### Sociodemographic variables

Demographic variables including age, gender, ethnicity, and social grade were taken using standard UK Office for National Statistics [17] measures.

#### Acceptability measures

Likert scale responses were developed in line with the seven Theoretical Framework of Acceptability (TFA) constructs used to assess acceptability [10]. The items were developed through consensus using the expertise within the research team, and in the absence of a suitable existing measure, psychometric properties of the measure were evaluated. Study-specific amendments were made to the items consistent with the VHS. Seven items were developed, e.g. “On a scale of 0–10, how much effort was required to use the volitional help sheet?” (burden; *no effort* [0]-*lots of effort* [10]). Item wordings were developed to closely resemble the definitions provided for each domain of the TFA [4, 10]. The items used to measure each TFA domain are presented in Supplementary File A. Participants were invited to complete the TFA measures at baseline and six-month follow-up.

Reliability was assessed using test-retest reliability (intra-class correlation coefficients). A series of two-way mixed-effects models with measures of absolute agreement were used. ICCs were determined as < 0.40 (poor), 0.40–0.75 (fair to good), and > 0.75 (excellent; [18]). Discriminant validity was assessed using inter-item correlations (Pearson’s *r*). Pearson’s correlation coefficient (*r*) was used to assess the strength of the relationship between the items. Pearson’s r is interpreted as 0.10 (small effect), 0.30 (medium effect), and 0.50 (large effect) (Cohen, 1988). A series of pairwise correlations were conducted to examine relationships between the seven TFA items. As each item is deemed to measure a different construct (*Affective Attitude, Burden, Ethicality, Self-efficacy, Opportunity costs, Intervention coherence*, and *Perceived effectiveness*), low correlation between items overall was expected (Pearson’s *r* < .50).

Test-retest reliability results are reported in Table 1. Data are analysed according to participants allocated to the control condition who completed each item at baseline and follow-up: *Affective Attitude* (*n* = 174), *Burden* (*n* = 170), *Ethicality* (*n* = 167), *Self-efficacy* (*n* = 170), *Opportunity Costs* (*n* = 153), *Intervention Coherence* (*n* = 167), *Perceived Effectiveness* (*n* = 168). Test–retest reliability was fair to good for all of the items (ICC 0.469-0.703).

**Table 1:** Reliability demonstrated by intra-class correlation coefficient (ICC) and 95% confidence intervals (CI) for TFA items (control group only)

Item	Reliability data	
	ICC	95% CI
Affective attitude (n = 345)	0.703**	0.428–0.639
Burden (n = 339)	0.563**	0.257–0.512
Ethicality (n = 331)	0.620**	0.484–0.720
Self-efficacy (n = 338)	0.624**	0.491–0.722
Opportunity costs (n = 306)	0.585**	0.430–0.699
Intervention coherence (n = 331)	0.469**	0.278–0.609
Perceived effectiveness (n = 332)	0.685**	0.573–0.768

** *p* < .001

Discriminant validity results for Time 1 are reported in Table 2. Fourteen of the correlations were small to medium (*r* = − .112–0.481). Three of the correlations were large: affective attitude and perceived effectiveness (*r* = .524), ethicality and perceived effectiveness (*r* = .590), and self-efficacy and intervention coherence (*r* = .604). Four of the correlations were non-significant. In contrast, at Time 2 (Table 2), eleven of the correlations were small to medium (*r* = .113–0.472). Six of the correlations were large: ethicality and affective attitude (*r* = .503), self-efficacy and affective attitude (*r* = .500), intervention coherence and self-efficacy (*r* = .657), perceived effectiveness and affective attitude (*r* = .590), perceived effectiveness and ethicality (*r* = .727), and perceived effectiveness and opportunity costs (*r* = .536). Three of the correlations were non-significant.

**Table 2:** Pearson’s correlations in relation to Time 1^a^ and Time 2^b^ data

Item	Affective Attitude	Burden	Ethicality	Self-efficacy	Opportunity costs	Intervention coherence	Perceived effectiveness	Time 1 M (SD)	Time 2 M (SD)
Affective Attitude	-	− 0.168**^1^	0.503**^3^	0.500**^3^	0.134*^1^	0.354**^2^	0.590**^3^	5.47 (2.22)	5.62 (2.38)
Burden	− 0.112*^1^	-	0.113*^1^	− 0.333**^2^	0.317**^2^	− 0.259**^1^	− 0.014	4.98 (2.86)	5.07 (3.00)
Ethicality	0.453**^2^	0.137**^1^	-	0.472**^2^	0.271**^1^	0.381**^2^	0.727**^3^	4.83 (2.66)	4.76 (2.72)
Self-efficacy	0.433**^2^	− 0.262**^1^	0.384**^2^	-	0.070	0.657**^3^		6.18 (2.62)	6.26 (2.91)
Opportunity costs	0.100	0.248**^1^	0.191**^1^	− 0.004	-	0.056	0.536**^3^	2.89 (2.66)	2.87 (2.92)
Intervention coherence	0.351**^2^	− 0.169**^1^	0.262**^1^	0.604**^3^	− 0.023	-	0.241**^1^	6.49 (2.66)	6.66 (2.79)
Perceived effectiveness	0.524**^3^	0.037	0.590**^3^	0.481**^2^	0.233**^1^	0.454**^2^	-	5.25 (2.57)	5.25 (2.79)

*Notes.* Correlations below the diagonal column refer to Time 1; correlations above the diagonal column refer to Time 2

*p < .05; **p < .01

^1^Small relationship; ^2^Medium relationship; ^3^Large relationship

### Analysis

Descriptive statistics were used to summarise sociodemographic variables. Chi-square was used to compare our sample of people who reported a previous history of self-harm with general population data collected as part of the Adult Psychiatric Morbidity Survey [19]. The success of the randomisation procedure was checked using MANOVA, and two-way repeated measures ANOVAs were used to assess the effect of the two versions of the volitional help sheet at six-month follow-up on acceptability ratings over time. MANOVA was used to establish whether the participants who remained in the study, or dropped out before follow-up were similar at baseline in terms of their demographic characteristics and their TFA ratings. Whilst the seven TFA domains are independent constructs, it may be possible that changes in one domain may affect other domains. Therefore, all TFA domains were included in the analyses, regardless of which domains the refinements were made in line with. This approach allows the researchers to determine whether making refinements in one TFA domain, could impact acceptability ratings of other TFA domains. The between-participants factor was *condition* (original volitional help sheet versus modified volitional help sheet). The within-participants factor was *time* (baseline and six-month follow-up acceptability scores). To determine whether floor or ceiling effects were observed, we calculated the proportion of participants scoring at each point on the rating scale of each item. The recognized value of 15% of the sample was used to determine whether floor and ceiling effects were observed with the proportion of responses being at either the minimum or maximum point of the items [20]. G power software [21] was used to calculate the required sample size. Assuming statistical power of 0.95 and an error probability of 0.05, to detect a small effect size (*d* = 0.20), the total required sample size for a fully powered randomized controlled trial was estimated to be *N* = 328.

## Results

### Sample characteristics

The final sample (*n* = 393) comprised mostly women (67.7%), and 23.9% were aged 18–34 years, 22.4% were aged 35–44 years, 20.4% were aged 45–54 years, and 33.3% were aged 55 years and older. The majority of the sample was White (92.1%), and 65.6% were of higher social grade (non-manual worker) (see Table 3). Characteristics of our sample closely resembled the characteristics of people who reported a history of self-harm according to the Adult Psychiatric Morbidity Survey of the general population [19] in terms of age. However, our sample contained a lower proportion of men, a higher proportion of people from a white background, and a lower proportion of people from a minority ethnic background, compared to national data.

**Table 3:** Sample demographics in the two groups (final sample)

Variable	Control condition (n = 199)		Experimental condition (n = 194)		Total (n = 393)		*General population data* ^*a*^	*X2 for difference between sample and population*
	n	%	n	%	n	%		
Gender								
Women	131	65.8	135	69.6	266	67.7	54.5	3.57 (*P* = .06)
Men	68	34.2	59	30.4	127	32.3	45.5	4.12 (*P* < .05)
Age								
18–34	51	25.6	43	22.2	94	23.9	26.4	0.11 (*P* = .74)
35–44	43	21.6	45	23.2	88	22.4	17.8	0.50 (*P* = .48)
45–54	40	20.1	40	20.6	80	20.4	21.1	0.03 (*P* = .86)
55+	65	32.7	66	34.0	131	33.3	34.6	0.09 (*P* = .77)
Ethnicity								
White	186	93.5	176	90.7	362	92.1	87.1	24.42 (*P* < .05)
BAME	6	3.0	8	4.1	14	3.6	12.9	5.21 (*P* < .05)
Prefer not to say	7	3.5	3	1.5	3	0.8		
Social grade								
Non-manual worker	129	64.8	129	66.5	258	65.6	-	-
Manual / unemployed	69	34.7	63	32.5	132	33.6	-	-
Not stated	1	0.5	2	1.0	3	0.8		

### Randomisation check

**Fig. 3:**
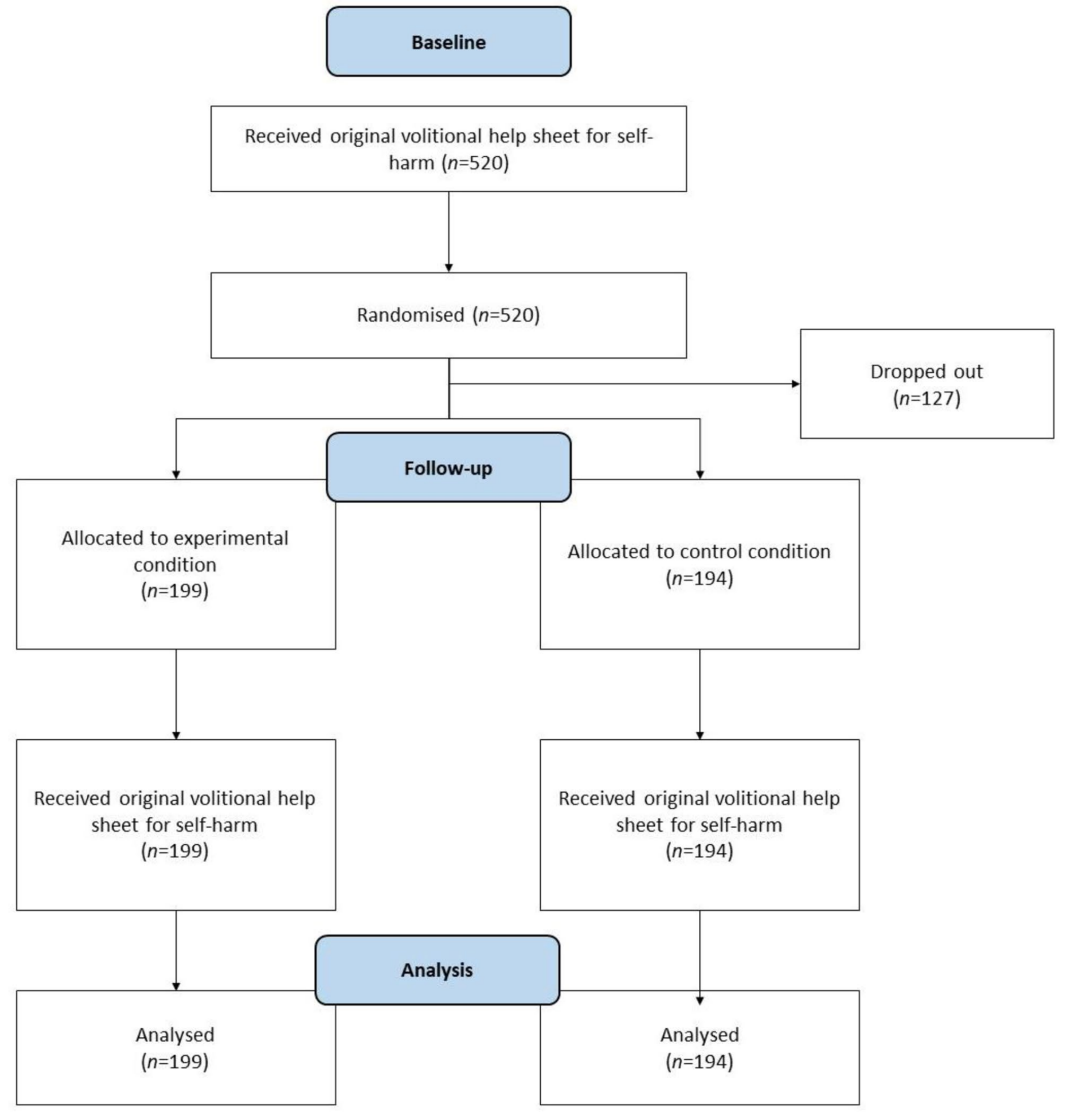
Flow diagram showing participant allocation to each condition

The success of the randomisation procedure was checked using MANOVA (a flow diagram is presented in Fig. 3). The independent variable was *condition* with two levels: modified intervention versus original intervention. The dependent variables were age, gender, and baseline measures of all seven TFA domains. The multivariate test, *F*(9, 319) = 0.97, *p* = .46, *η*_p_^2^ < 0.03, and all the univariate tests, *F*s(1, 327) = 0.02 to 1.7, *p*s > 0.19, *η*_p_^2^ = 0.01, were nonsignificant, indicating successful randomisation.

### Baseline equivalence check

Equivalence between the participants who dropped out versus remained in the study with respect to baseline characteristics was checked using MANOVA. The independent variable was *completed at follow-up with* two levels: yes versus no. The dependent variables were age, gender, and baseline measures of all seven TFA domains. The multivariate test, *F*(9, 423) = 0.81, *p* = .61, *η*_p_^2^ = 0.02, and all the univariate tests, *F*s(1, 431) = 0.00 to 2.3, *p*s > 0.13, *η*_p_^2^ s < 0.01, were nonsignificant, indicating no differences in demographic characteristics of TFA ratings.

### Changes in acceptability ratings over time

The proportion of participants scoring at each point on the rating scale of each item at baseline and follow-up is presented in Table 4. There are two key findings. First, we observed ceiling effects for the intervention coherence item at baseline (16.4%) and follow-up (18.9%). Second, there was a high proportion of responses at the lower end of the opportunity costs item at baseline (33.6%) and follow-up (37.6%). Table 5 shows the results of the repeated measures ANOVA to assess changes in acceptability ratings over time. The main effects of time and condition on acceptability scores were nonsignificant for all TFA domains (see Table 5). Similarly, all time *x* condition interaction effects were nonsignificant (all *η*_p_^2^ < 0.01).

**Table 4:** Proportion of responses at each point according to item at baseline and follow-up

Item												Total responses	Missing
Affective attitude	0	1	2	3	4	5	6	7	8	9	10		
Baseline	18 (3.7)	8 (1.7)	21 (4.4)	37 (7.7)	29 (6.0)	174 (36.2)	44 (9.1)	56 (11.6)	40 (8.3)	22 (4.6)	32 (6.7)	481	39
Follow-up	12 (3.2)	6 (1.6)	13 (3.5)	31 (8.4)	24 (6.5)	130 (35.1)	30 (8.1)	44 (11.9)	35 (9.5)	9 (2.4)	36 (9.7)	370	150
Burden	0	1	2	3	4	5	6	7	8	9	10		
Baseline	49 (10.3)	21 (4.4)	28 (5.9)	44 (9.2)	25 (5.3)	84 (17.6)	53 (11.1)	69 (14.5)	56 (11.8)	25 (5.3)	22 (4.6)	476	44
Follow-up	42 (11.5)	20 (5.5)	27 (7.4)	22 (6.0)	23 (6.3)	58 (15.8)	39 (10.7)	49 (13.4)	44 (12.0)	14 (3.8)	28 (7.7)	366	154
Ethicality	0	1	2	3	4	5	6	7	8	9	10		
Baseline	40 (8.5)	21 (4.5)	45 (9.6)	49 (10.4)	29 (6.2)	114 (24.3)	40 (8.5)	53 (11.3)	39 (8.3)	20 (4.3)	20 (4.3)	470	50
Follow-up	38 (10.5)	18 (5.0)	22 (6.1)	27 (7.5)	30 (8.3)	100 (27.7)	40 (11.1)	27 (7.5)	27 (7.5)	9 (2.5)	23 (6.4)	361	159
Self-efficacy	0	1	2	3	4	5	6	7	8	9	10		
Baseline	14 (3.0)	18 (3.8)	16 (3.4)	24 (5.1)	32 (6.8)	105 (22.2)	40 (8.5)	63 (13.3)	64 (13.5)	35 (7.4)	62 (13.1)	473	47
Follow-up	24 (6.6)	7 (1.9)	16 (4.4)	20 (5.5)	23 (6.3)	45 (12.3)	34 (9.3)	54 (14.8)	50 (13.7)	33 (9.0)	59 (16.2)	365	155
Opportunity costs	0	1	2	3	4	5	6	7	8	9	10		
Baseline	149 (33.6)	37 (8.3)	23 (5.2)	43 (9.7)	18 (4.1)	109 (24.5)	19 (4.3)	16 (3.6)	15 (3.4)	9 (2.0)	6 (1.4)	444	76
Follow-up	130 (37.6)	26 (7.5)	23 (6.6)	24 (6.9)	14 (4.0)	75 (21.7)	11 (3.2)	19 (5.5)	6 (1.7)	8 (2.3)	10 (2.9)	349	174
Intervention coherence	0	1	2	3	4	5	6	7	8	9	10		
Baseline	15 (3.2)	10 (2.1)	16 (3.4)	25 (5.3)	25 (5.3)	85 (18.1)	47 (10.0)	58 (12.3)	78 (16.6)	34 (7.2)	77 (16.4)	470	50
Follow-up	20 (5.6)	5 (1.4)	9 (2.5)	14 (3.9)	17 (4.7)	50 (13.9)	36 (10.0)	51 (14.2)	48 (13.3)	42 (11.7)	68 (18.9)	360	160
Perceived effectiveness	0	1	2	3	4	5	6	7	8	9	10		
Baseline	40 (8.5)	10 (2.1)	29 (6.1)	31 (6.6)	25 (5.3)	118 (25.0)	64 (13.6)	61 (12.9)	56 (11.9)	16 (3.4)	22 (4.7)	472	48
Follow-up	33 (9.2)	11 (3.1)	27 (7.5)	25 (6.9)	21 (5.8)	66 (18.3)	46 (12.8)	48 (13.3)	46 (12.8)	13 (3.6)	24 (6.7)	360	160

**Table 5:** Two-way repeated measures ANOVAs were used to assess the effect of the two versions of the volitional help sheet on acceptability ratings over time

Dependent Variables	Baseline		Follow-up		*Time*		*Condition*				time*condition		
	*M*	*SD*	*M*	*SD*	*F*	*p*	η_p_^2^	*F*	*p*	η_p_^2^	*F*	*p*	η_p_^2^
Affective Attitude					2.91	0.09	0.01	1.03	0.31	0.00	0.67	0.41	0.00
Control group	5.31	2.33	5.63	2.36									
Experimental group	5.63	2.15	5.74	2.43									
Burden					0.69	0.41	0.00	0.14	0.71	0.00	0.80	0.37	0.00
Control group	5.03	2.87	5.02	2.98									
Experimental group	4.78	2.90	5.08	2.96									
Ethicality					0.21	0.65	0.00	0.49	0.49	0.00	0.33	0.56	0.00
Control group	4.88	2.65	4.90	2.75									
Experimental group	4.79	2.68	4.63	2.67									
Self-efficacy					0.23	0.64	0.00	0.76	0.39	0.00	0.02	0.90	0.00
Control group	6.34	2.62	6.43	3.05									
Experimental group	6.13	2.62	6.18	2.78									
Opportunity Costs					0.00	0.96	0.00	1.49	0.22	0.01	2.14	0.15	0.01
Control group	2.86	2.80	2.59	2.86									
Experimental group	2.92	2.48	3.17	2.97									
Intervention Coherence					2.59	0.11	0.01	0.05	0.83	0.00	1.45	0.23	0.00
Control group	6.63	2.65	6.70	2.72									
Experimental group	6.38	2.71	6.84	2.69									
Perceived Effectiveness					0.13	0.71	0.00	0.03	0.86	0.00	0.30	0.59	0.00
Control group	5.18	2.72	5.31	2.66									
Experimental group	5.30	2.43	5.28	2.87									

## Discussion

This paper aimed to operationalise an approach to intervention acceptability that: (a) ensured that a large representative sample of people with lived experience is involved in the process of improving intervention acceptability; and (b) adopted a theory-driven, experimental approach to evaluating whether refining an intervention based on participant feedback increases acceptability. To our knowledge, this is the first study to apply the TFA to develop an experimental approach to assess whether incorporating participant feedback brings about improvements in intervention acceptability.

Although the number of participants exceeded the required numbers based on prior power calculations, there were no statistically significant differences in acceptability ratings between the control group (the original intervention) and the intervention group (a modified version of the volitional help sheet based on participant feedback) at six-month follow-up. Consequently, more changes may be required to the modified intervention to further increase acceptability. Future research should therefore aim to build on our findings and examine how making further adaptations may affect perceptions of acceptability, with a focus on two specific domains: *opportunity costs* and *intervention coherence*. There are two possible reasons that may explain our null findings. First, we observed a high proportion of responses at the lower end of the opportunity costs item (i.e. perceptions of what is given up, such as time, to take part in an intervention) at baseline and follow-up. This may suggest that a key area for refinement would be to explore ways of reducing the perceived costs associated with engaging in an intervention, such as providing incentives for participation [22]. Second, we observed a high proportion of responses at the higher end of the intervention coherence item at baseline and follow-up. Whilst this may suggest the instructions accompanying the original intervention as well as the changes we made to our intervention at follow-up were both perceived to be clear and well understood by participants, further acceptability research within this domain would help to ensure that the possibility of ceiling effects could be eliminated.

### Implications

Incorporating participant feedback in the design of interventions is an important stage of intervention development [1], such as feedback about content, format, style and delivery of interventions [7], yet this is still an emerging field [23]. Most intervention acceptability research to-date typically relies on relatively small sub-samples of people with limited focus on actual experiences of engaging with an intervention, leaving judgements open to the “third-person effect” (i.e. perceiving an intervention as having a greater effect on others than on himself or herself) [24]. Informed by the TFA we have developed a process for researchers to systematically examine ways of improving intervention acceptability as part of iterative cycles of intervention development. However, further refinements are needed to our process to ensure our measure is sensitive enough to identify changes over the long-term. Whilst we observed no significant changes in acceptability ratings at follow-up, future intervention development research must aim to deploy appropriate measures to ensure that changes made in response to participant and public feedback actually do increase intervention acceptability.

### Strengths and limitations

Previous studies applying the TFA to explore intervention development have primarily been qualitative studies [25, 26]. Few studies have deployed the TFA as a quantitative measure [10, 27]. For example, Renko et al. assessed the acceptability of a training programme to enhance teachers’ physical activity promotion. Whilst outside the scope of the present study, it would be valuable to explore the views of participants who dropped out of the study at baseline, and consequently did not provide feedback at follow-up on the views of the refined intervention. To our knowledge, the present research is the first time a study has developed an experimental methodology, based on the TFA, to evaluate whether changes made to an intervention based on participant feedback improves acceptability. Our measure is designed to assess each of the seven TFA domains: *affective attitude, burden, perceived effectiveness, ethicality, intervention coherence, opportunity costs*, and *self-efficacy*. This is important because having a systematic method of determining how to increase intervention acceptability, according to the different facets associated with acceptability, also allows researchers to examine whether processes designed to increase acceptability of interventions are effective following refinements to the intervention.

Increasing acceptability is important when developing interventions iteratively, and in line with recognised guidance for intervention development. We present an experimental approach that allows researchers to target specific TFA domains in future iterations of interventions (e.g. addressing perceived effort to engage with interventions). Another strength of our process is the involvement of stakeholders in the design and modification of interventions, allowing participants to provide specific feedback on an existing intervention with the overarching aim of increasing intervention acceptability and feasibility [1].

## Conclusions

Incorporating participant feedback is an important consideration for the design, implementation and evaluation of interventions. The Theoretical Framework of Acceptability provides a framework designed to systematically examine the seven components associated with acceptability. Our tool provides a systematic method of evaluating whether refining an intervention based on participant feedback increases acceptability. With further testing across different populations and interventions we hope the tool provides researchers with a method of improving intervention acceptability.

### Electronic supplementary material

Below is the link to the electronic supplementary material.

Supplementary Material 1

## Data Availability

The datasets used and/or analysed during the current study are available from the corresponding author on reasonable request.
