# Supplementary material for: Does refining an intervention based on participant feedback increase acceptability? An experimental approach
Source: BMC Public Health. 2023 Aug 22;23:1598. doi: 10.1186/s12889-023-16344-w (PMC10463387; doi:10.1186/s12889-023-16344-w)
Supplement: Supplementary file 1 — Supplementary Material 1 [file 12889_2023_16344_MOESM1_ESM.docx]

Supplementary file A: The Theoretical Framework of Acceptability domains

| Domain | Question |
| --- | --- |
| Affective attitude | On a scale of 0-10, how good or bad did you feel when using the volitional help sheet? |
| Burden | On a scale of 0-10, how much effort was required to use the volitional help sheet? |
| Ethicality | On a scale of 0-10, how much was using the volitional help sheet a good fit with your personal values? |
| Self-efficacy | On a scale of 0-10, how confident were you about using the volitional help sheet? |
| Opportunity | On a scale of 0-10, to what extent did you give up any benefits, profits, or values when using the volitional help sheet? |
| Intervention coherence | On a scale of 0-10, how confident were you that you understood the volitional help sheet and how it works? |
| Perceived effectiveness | On a scale of 0-10, how confident were you that the volitional help sheet is likely to achieve its purpose? |
